# Immune checkpoint inhibitor-induced endocrinopathies: a possible indicator of improved survival

**DOI:** 10.20945/2359-3997000000654

**Published:** 2023-06-19

**Authors:** Mariana Ornelas, Marta Borges-Canha, Pedro Gouveia, Margarida Ferreira, Eduarda Resende, Maritza Sá, Silvestre Abreu

**Affiliations:** 1 Hospital Central do Funchal Departamento de Endocrinologia Madeira Portugal Departamento de Endocrinologia do Hospital Central do Funchal, Madeira, Portugal; 2 Centro Hospitalar Universitário de São João Departamento de Endocrinologia Porto Portugal Departamento de Endocrinologia do Centro Hospitalar Universitário de São João, Porto, Portugal

**Keywords:** Immune checkpoint inhibitors, immunotherapy, endocrine system diseases, drug-related side effects and adverse reactions

## Abstract

**Objective:**

To evaluate the association between the patients’ characteristics and the development of endocrine toxicity and to assess the association between endocrine-related adverse effects (ERAE) development and mortality.

**Subjects and methods:**

A retrospective observational study was conducted in 98 patients submitted to immunotherapy in our centre since its introduction in 2015 until March 2021. We excluded patients for which data regarding the corticotroph axis evaluation was missing. We used linear and logistic regression models to address our aims.

**Results:**

We observed a significant negative association between ERAE development and death (OR 0.32; p = 0.028). We detected no associations between ERAE and the following characteristics: age at immune checkpoint inhibitors (ICI) initiation, sex, diabetes mellitus, medical history, immunotherapy duration and ICI type.

**Conclusion:**

The development of an ERAE may be associated with a better overall survival rate in advanced oncologic disease, supporting the role of an unleashed immune system response to malignant cells.

## INTRODUCTION

Immune checkpoint inhibitors (ICI) are a recent oncologic treatment used in patients with locally advanced or metastatic neoplasms with significant improvements in clinical outcomes and survival rates (1). These drugs are known as immunotherapy and consist of monoclonal antibodies targeted to specific immune checkpoints that enable antitumoral immune system response, thus decreasing immune tolerance to the tumour and promoting cell death. These antibodies may be directed to various molecules, such as cytotoxic T-lymphocyte antigen-4 (anti-CTLA-4) – ipilimumab, programmed death 1 (anti-PD-1) – nivolumab and pembrolizumab, or its ligand (anti-PD-L-1) – atezolizumab and durvalumab (2).

With the increased use of immunotherapy in the last decade, a wide variety of immune-related adverse events (IRAE) has been reported. Specifically, endocrine-related adverse events (ERAE) are thought to occur in about 10% of cases. Thyroid dysfunction and hypopituitarism are the most common endocrinopathies described; additionally, primary adrenal insufficiency, type 1 diabetes and primary hypoparathyroidism can also occur but less commonly. The spectrum of endocrine toxicities can be categorized as mild (grade 1) to death (grade 5) following the Common Terminology Criteria for Adverse Events (CTCAE) developed by the National Cancer Institute (1).

Currently, there are no established predictive markers for ERAE. Additionally, ERAE development may be associated with better overall cancer survival rates resulting from immune system enhancement (3).

This study evaluated the association between some patient clinical characteristics and the development of endocrine toxicity and assessed whether ERAE occurrence is related to mortality.

## SUBJECTS AND METHODS

### Study design and participants

We included all patients submitted to immunotherapy with anti-CTLA-4 (ipilimumab), anti-PD1 (nivolumab and pembrolizumab) and anti-PDL1 (atezolizumab and durvalumab) for advanced or metastatic tumours followed in our centre between its first introduction in 2015 until March 2021. Patients for which data were missing for the corticotroph axis evaluation were excluded from the analysis. Past systemic corticosteroid use was considered acceptable if discontinued more than 4 weeks prior to inclusion in study. Formal consent was not required for this type of study. Ethical approval for this study was obtained from *Comissão de Ética para a Saúde e da Comissão Científica e de lnvestigação do Serviço de Saúde da Região Autónoma da Madeira*, EPERAM, (S.22005586).

### Clinical parameters evaluated

The following parameters were evaluated: age at the beginning of therapy, sex, previous history of diabetes, cancer type, ICI type used, treatment duration, death, ERAE development (primary thyroid dysfunction, hypopituitarism, primary adrenal insufficiency and diabetes), ERAE time of onset after ICI initiation, endocrine toxicity grade and overall survival rate after ICI initiation.

The ERAE were defined as new biochemical abnormalities of the endocrine system with the application of usual diagnosis criteria and which were not previously documented in patients. Primary hypothyroidism was defined as a thyroid-stimulating hormone (TSH) above the upper limit of our laboratorial reference range and included the surge of overt hypothyroidism – free thyroxine (FT4) below the lower limit of normal – and subclinical hypothyroidism – FT4 within the normal laboratorial range (4). Thyrotoxicosis was defined by a TSH value below laboratorial reference range and included overt hyperthyroidism – with elevated FT4 – and subclinical hyperthyroidism - with normal FT4 (5).

Primary adrenal insufficiency was defined by a morning cortisol level below 5 μg/dL and plasmatic adrenocorticotropic hormone (ACTH) over twice the upper limit of the reference range (6).

Hypopituitarism was defined as the presence of at least one pituitary hormonal deficiency, with the following criteria: adrenal insufficiency was defined as a morning cortisol < 5 μg/dL and an ACTH value below the normal laboratorial range. Hypogonadism was diagnosed when follicle-stimulating hormone (FSH) or luteinizing hormone (LH) were below or within the lower end of the reference range combined with low serum testosterone levels in males and low serum estrogen levels in females. A low FT4 in conjunction with a low or normal TSH defined central hypothyroidism. GH deficiency was defined by a low specific age IGF-1 value and the presence of other pituitary hormone deficiencies (7). Diabetes was defined as fasting plasma glucose ≥126 mg/dL, glycated haemoglobin ≥ 6.5%, 2 h plasma glucose after a 75-g oral glucose tolerance test ≥ 200 mg/dL, or the use of antihyperglycemic drugs (8). The grade of ERAE was categorized following the National Cancer Institute's Common Terminology Criteria for Adverse Events, version 5 (9).

### Statistical analysis

Continuous variables are described as mean ± standard deviation (SD) and categorical variables as proportions (percentages). We performed unadjusted linear and logistic regression models to address the potential associations between ERAE and the patients’ characteristics, and between ERAE and death. Two-sided P values less than 0.05 were considered statistically significant. Statistical analyses were performed with Stata software, version 14.1 (StataCorp).

## RESULTS

### Baseline population characteristics

A total of 94 patients were included in this analysis (Table 1). Mean age at ICI initiation was 60.4 ± 11.5 years, of which 76.6% were males (*n* = 72). About 14.9% (*n* = 14) of patients had diabetes mellitus. The most frequent cancer type was non-small-cell lung carcinoma (NSCLC) (47.8%, *n* = 45) followed by melanoma (25.5%, *n* = 24), renal cancer (10.6%, *n* = 10), head and neck cancer (6.4%, *n* = 6), urologic cancer (4.3%, *n* = 4), Hodgkin's lymphoma (3.2%, *n* = 3), gastrointestinal cancer (1.1%, *n* = 1) and breast cancer (1.1%, *n* = 1). Most patients (76.6%, *n* = 72) were treated with anti-PD-1 therapy (nivolumab or pembrolizumab) followed by anti-PDL-1 therapy (19.1%, *n* = 18) and anti-CTLA-4 therapy (4.3%, *n =* 4). The median treatment duration was 85 (0.0-180.0) days. At the end of this study analysis, we found a mortality rate of 61.7% (*n* = 58).

**Table 1:** Baseline population characteristics

		n = 94
Age at ICI initiation, years		
	Mean (SD)	60.4 (±11.5)
	Male sex, n (%)	72 (76.6)
DM, n (%)		14 (14.9)
Cancer type, n (%)		
	NSCLC	45 (47.8)
	Melanoma	24 (25.5)
	Renal	10 (10.6)
	Head and neck	6 (6.4)
	Urologic	4 (4.3)
	Hodgkin's lymphoma	3 (3.2)
	Gastrointestinal	1 (1.1)
	Breast	1 (1.1)
ICI type, n (%)		
	Anti-CTLA-4^a^	4 (4.3)
	Anti-PD1^b^	72 (76.6)
	Anti-PDL1^c^	18 (19.1)
Treatment duration, days		
	Median (IQR)	85 (0.0-180.0)
	Deaths, n (%)	58 (61.7)

Abbreviations: CTLA, cytotoxic T-lymphocyte antigen; DM, diabetes mellitus; ICI, immune checkpoint inhibitor; IQR, interquartile range; NSCLC, non-small cell lung cancer; PD, programmed death; PD-L, programmed death ligand; SD, standard deviation.

a Anti-CTLA-4 treatment: ipilimumab

b Anti-PD-1 treatment: nivolumab or pembrolizumab

c Anti-PDL-1 treatment: atezolizumab or durvalumab

#### Endocrine-related adverse events (ERAE): characterization

A total of 22 ERAE (23.4%) were documented over the median treatment duration of 85 days, as summarized in Table 2. The most common endocrine disease was thyroid dysfunction, which occurred in 63.64% (*n* = 14) of patients submitted to ICI. Subclinical hypothyroidism (22.73%, *n* = 5) and thyroiditis (22.73%, *n* = 5) were the most frequently reported thyroid dysfunction. Hypopituitarism appeared in 36.36% (*n* = 8) of patients and the corticotroph axis was the most affected lineage. None of the patients developed diabetes or primary adrenal insufficiency. The median time of onset after ICI initiation was 87 days (IQR 58.5-145.0). Grade 1 ERAE were the most reported in 72.7% of patients (*n* = 16) followed by grade 2 in 27.3% of patients (*n* = 6). There were no life-threatening complications observed. The median overall survival rate was 164.5 (92.0-290.0) days after ICI initiation.

**Table 2:** Endocrine-related adverse events characterization

ERAE development, n (%)		22 (100)
Primary thyroid dysfunction, n (%)		14 (63.64)
	Subclinical hypothyroidism	5 (22.73)
	Overt hypothyroidism	3 (13.64)
	Thyroiditis	5 (22.73)
	Thyrotoxicosis	1 (4.55)
Hypopituitarism, n (%)		8 (36.36)
	Corticotroph axis only	5 (22.73)
	Corticotroph axis plus gonadotroph axis	3 (13.64)
Primary adrenal insufficiency, n (%)		0 (0)
Diabetes, n (%)		0 (0)
ERAE time of onset after ICI initiation, days		
	Median (IQR)	87 (58.5-145.0)
ERAE grade, n (%)		
	1	16 (72.7)
	2	6 (27.3)
Overall survival rate after ICI initiation, days		
	Median (IQR)	164.5 (92.0-290.0)

Abbreviations: ERAE, endocrine-related adverse events; ICI, immune checkpoint inhibitor; IQR, interquartile range.

#### Endocrine-related adverse events according to ICI type

Table 3 presents endocrine complications according to ICI type used. Primary thyroid dysfunction developed secondary to anti-PD-1 use in 78.6% (*n* = 11) of patients and secondary to anti-PDL-1 use in 21.4% (*n* = 3) of patients. All cases of hypopituitarism (*n* = 8) were due to *anti-PD-1* use. There were no endocrine complications registered with anti-CTLA-4 use.

**Table 3:** Endocrine-related adverse events according to ICI type

Primary thyroid dysfunction, n (%)		14 (100)
	Anti-PD-1^a^	11 (78.6)
	Anti-PDL-1^b^	3 (21.4)
Hypopituitarism, n (%)		8 (100)
	Anti-PD-1^a^	8 (100)

Abbreviations: ICI, immune checkpoint inhibitor; PD, programmed death; PD-L, programmed death ligand.

a Anti-PD-1 treatment: nivolumab or pembrolizumab

b Anti-PDL-1 treatment: atezolizumab or durvalumab

#### Association of the patients’ characteristics and ERAE development

Table 4 summarizes the association between various patient characteristics and ERAE development. No association between ERAE and age at ICI initiation, sex, DM history, immunotherapy duration and ICI type was found.

**Table 4:** Association between patients’ characteristics and ERAE development

Variable	ERAE development	
	OR	P value
Age at ICI initiation	1.04 (0.99-1.09)	0.151
Sex	0.51 (0.13-1.94)	0.324
DM	1.01 (0.25-4.04)	0.988
Treatment duration	0.99 (0.99-1.00)	0.624
Death	0.32 (0.12-0.89)	0.028
**Variable**	**β**	**P value**
ICI type	0.01 (-1.01,1.08)	0.991

Abbreviations: DM, diabetes mellitus; ERAE, endocrine related adverse events; ICI, immune checkpoint inhibitor; OR, Odds ratio.

#### Association of ERAE development and mortality

A significant decrease in the risk of death with ERAE development (OR 0.32; p = 0.028) was documented, as presented in Table 5.

**Table 5:** Association between ERAE development and mortality

Variable	Death	
	OR	P value
ERAE development	0.32 (0.12-0.89)	0.028

Abbreviations: ERAE, endocrine related adverse events; OR, Odds ratio.

## DISCUSSION

This is a retrospective study that studied various patient characteristics and their association with ERAE development and assess whether endocrine toxicity was related to mortality. We did not find any association between the patient clinical characteristics studied and ERAE development. We did, however, report a significant negative association (OR 0.32; p = 0.028) between the development of endocrine toxicities secondary to ICI use and patient mortality.

Our results suggest that the development of an ERAE may be linked to a higher overall survival (OS) rate of patients with advanced or metastatic cancer. This association seems to be related to the antitumoral response driven by an unleashed immune system response empowered by immunotherapy. Labadzhyan and cols. (10) acknowledged similar results, reporting higher overall survival among patients who developed endocrinopathies secondary to ICI compared to those who did not develop any ERAE, even though the authors advise caution in the interpretation of the results, considering the heterogeneity of the neoplasms and ICI types used. Furthermore, this association may be extended to other affected organs, suggesting that the immune system is erratically triggered in a multisystem way. To corroborate this finding, a systematic review and meta-analysis conducted by Hussaini and cols. (11) showed a positive association between the development of an IRAE and OS in patients treated with ICIs, irrespective of disease site, type of ICI used and IRAE. There are some conflicting results reported in the literature in regard to the association between the IRAE grade and improved OS. Hussaini and cols. (11) reported worse OS in the face of severe adverse event development (grade 3 or higher). Nevertheless, some studies suggest that the development of more severe toxicities may be associated with better cancer prognosis, supporting the view that all-grade IRAE may be a potential prognostic biomarker of therapy response (12,13). Notably, the majority of ERAE are reported as mild to moderate in severity, and our results were in line with those findings (grade 1 to 2) (14). Interestingly, the current association between IRAE secondary to ICI use and a lower mortality may be stronger in accordance with the affected toxicity site. Endocrine dysfunction is related to significant survival benefits in patients submitted to ICI in parallel with skin and gastrointestinal tract adverse events rather than toxicities occurring in liver and lung (15–17). Particularly, thyroid dysfunction has been linked to anti-tumour effects of ICI and was proposed as a surrogate marker for clinical response (18–19). Currently, the establishment of predictive factors of ERAE development is still in progress, with few conclusions. Some authors defend that older age, male sex treatment duration and the presence of endocrine-specific autoantibodies may be associated with ERAE development (10). Another significant question that is being addressed in the current literature is whether ERAE predictors may possibly be used as predictors of immunotherapy efficacy alongside its adverse events (20). It is of the greatest interest to develop simple and accurate predictive tools of ERAE secondary to ICI use to support prompt diagnosis and individualize patient management. There are some limitations of this study that must be acknowledged. The mortality rate assessed in this study did not account for the cancer type and the ICI used, so the overall survival rate must be interpreted with caution due to the heterogeneity of these features. Some patients included in the study (n = 4) had a previous history of other cancer treatment (tyrosine kinase inhibitor), which may have influenced the thyroid toxicity surge. Nevertheless, the patients’ thyroid function at therapy initiation was normal. The low sample size of the study's population may have interfered with the lack of association between the patients’ clinical characteristics and ERAE occurrence. Another limitation of the study is the retrospective observational design, which may be subject to some biases.

In conclusion, this study related a negative association between the ERAE development and mortality rate, thus suggesting a better overall survival rate in patients with locally advanced or metastatic cancer who develop endocrine toxicity. Our study documented mild to moderate ERAE (grade 1 to 2). These findings are consistent with other previously reported results, supporting the view that the development of a mild to moderate ICI toxicity is paradoxically associated with its efficacy.
